# State estimation of multi-sensor systems based on error-state Kalman

**DOI:** 10.1371/journal.pone.0338917

**Published:** 2025-12-26

**Authors:** Yang Liu, Peng Liu, Yu Shi

**Affiliations:** Department of Communication Electronic Countermeasure, Aviation University of Air Force, Changchun, China; Beijing Institute of Technology, CHINA.

## Abstract

With the rapid advancement of multi-sensor systems, the capabilities of robots in complex scenes are gradually improving. A multi-sensor system state estimation algorithm based on error-state Kalman filter is proposed to address the issues of noise interference, sensor data loss, and interference from moving targets in dynamic scenes. Firstly, the state estimation method of multi-sensor system based on sequential fusion framework is designed to effectively integrate and process different sensor data. On this basis, further research is conducted to design a lightweight detection algorithm based on multi-sensor data for identifying and processing moving targets in dynamic scenes, thereby reducing their interference with state estimation. Finally, a sequential fusion odometer based on error-state Kalman filtering is constructed to enhance the accuracy and stability of state estimation, and further optimize the performance of the entire state estimation system. Experimental results show that the proposed algorithm achieves an estimation error of only 0.36, significantly outperforming the comparison algorithms. The mean average precision on the KITTI and NuScenes datasets reaches 0.89 and 0.85, respectively. The algorithm maintains stable efficiency across varying data scales, with low packet loss rates and controllable false detection rates in medium-to-long-term state estimation. Packet loss rates in noisy environments and dynamic target interference scenarios are 0.53% and 1.07%, respectively. The multi-sensor fusion state estimation algorithm proposed in the study can effectively handle the interference problem in dynamic scenes, significantly improving the localization and mapping performance of robots in complex environments. The research provides an effective solution for the stable positioning and mapping of robots in complex dynamic environments, which is of great significance for improving the application performance of robots in fields such as autonomous driving and special operations.

## 1 Introduction

With the widespread application of robot technology in complex dynamic environments, the importance of state estimation in multi-sensor systems is becoming increasingly prominent [1,2]. In dynamic scenarios, robots face many challenges, such as noise interference, sensor data loss, and interference from moving targets. Accurately estimating the robot’s state and achieving stable positioning and mapping in complex environments is a key prerequisite for ensuring the efficient operation of robots in fields such as autonomous driving and special operations [3,4]. Multi-sensor systems involve various types of sensors, such as LiDAR, visual sensors, and inertial measurement units, among which there are nonlinear correlations and dynamic coupling relationships between sensor data [5]. Traditional state estimation methods, such as the Extended Kalman Filter (EKF), often face problems such as insufficient estimation accuracy and poor stability when dealing with high-dimensional, nonlinear, and dynamically changing sensor data.

Error-State Kalman Filter (ESKF) is a special Kalman filtering algorithm mainly used to deal with error correction problems in system state estimation, and is broadly utilized to enhance the accuracy and stability of system state estimation [6]. For example, Y. Wang et al. proposed a method based on a trackless Kalman filter and interactive multi-model approach to enhance vehicle path planning capabilities. Experimental results validated its high accuracy and adaptability [7]. Scholars such as D. He proposed a real-time state estimation method based on ESKF to address the problem of difficulty in uniformly generalizing the state evolution of robots on manifolds. Experimental results showed that the proposed method could simplify calculations while improving performance and computational efficiency [8]. T. Chang et al. proposed a state estimator based on variational Bayesian and ESKF for real-time description of robot motion estimation to solve the problem of difficulty in describing robot states due to dynamic influences. Experiments showed that the performance of the proposed state estimator was improved and the optimization effect was good [9]. Z. Wang et al. designed an odometer system based on the sliding window method using ESKF to achieve more accurate state estimation. The findings showed that the robustness of the proposed system was enhanced [10]. D. Shen et al. addressed the shortcomings of existing micro-short-circuit fault diagnosis methods by proposing an adaptive Kalman filter-based approach. This method diagnosed faults by calculating online short-circuit resistance, with experiments verifying its rapid convergence capability [11]. It can be seen that the relevant research of ESKF mostly focuses on single sensor error correction, which does not solve the dual problems of inability to correct errors when data is lost in multi-sensor dynamic scenarios and observation distortion caused by dynamic target interference. Moreover, it does not establish a quantitative correlation with target detection technology, making it difficult to distinguish the robot’s own motion error from external interference error [12].

You Only Look Once version 5 (YOLOv5) is a version of the You Only Look Once (YOLO) series object detection model, which has been widely used in multiple fields due to its real-time performance, lightweight, and high efficiency. For example, X. Liang et al. designed a detection network based on YOLOv5 to achieve more streamlined object detection. Findings denoted that the performance of the proposed detection network was significantly improved [13]. J. Han et al. proposed a lightweight image detection system based on Light-YOLOv5 to improve the accuracy of object detection. The experiment showed that the performance and efficiency of the proposed detection system were significantly improved, and the two were well balanced [14]. It can be seen from this that existing target detection and filtering methods only use detection results to simply eliminate dynamic area data, do not establish a quantitative correlation between detection semantics and filtering error models, and do not solve the multi-sensor time asynchronous problem [15].

In summary, existing filtering methods cannot accurately identify the source of errors, and the error correction direction deviates, making it difficult to meet the needs for high-precision estimation in dynamic scenarios. However, existing target detection methods lack a quantitative coordination mechanism with the filtering system and cannot convert the detected dynamic target characteristics (such as movement speed and occlusion rate) into quantitative parameters for filtering error correction. The detection results cannot effectively guide filtering optimization, which limits the performance of the overall system in dynamic scenes. In addition, existing fusion methods lack the design of dynamic target perception and fusion and cannot solve the allocation problem under dynamic interference [16]. Compared with existing parallel fusion (which requires synchronization of all sensor data, and is prone to overall failure due to single sensor packet loss) and centralized fusion (which has high computational complexity and is difficult to adapt to embedded devices), Sequential Fusion (SF) can support the orderly access and information integration of multiple types of sensors, fill data gaps through packet loss compensation, and can also dynamically adjust the observation weight of each sensor to distinguish the robot’s own motion errors and dynamic interference errors, achieve accurate attribution correction, and avoid low-precision data interference [17,18]. In addition, You Only Look Once version 5s (YOLOv5s) significantly reduces computational complexity by simplifying the model structure and reducing the number of parameters while ensuring a certain level of detection accuracy. It dynamically allocates detected dynamic target features and combines static target anchor points to assist packet loss completion to adapt to complex dynamic scenarios. Therefore, research is conducted on state estimation of multi-sensor systems based on SF, and use YOLOv5s adaptive accuracy adjustment and ESKF parallel calculation to quantify parameters and perform filtering corrections to achieve a balance between accuracy and real-time performance. Ultimately, a multi-sensor system state estimation method is constructed based on error-state Kalman. The research aims to effectively integrate multi-sensor data and improve state estimation accuracy and stability. The innovations of this study are: (1) establishing a quantitative mapping relationship between detection semantics and filtering errors through the dynamic weight allocation mechanism of SF; (2) designing an integrated process for multi-sensor spatio-temporal alignment and packet loss compensation to break through the limitations of data asynchrony and loss; (3) proposing a collaborative optimization strategy for lightweight detection and filtering calculations to balance accuracy and real-time performance.

## 2 Research design

### 2.1 State estimation of multi-sensor systems based on sequential fusion

**Fig 1:**
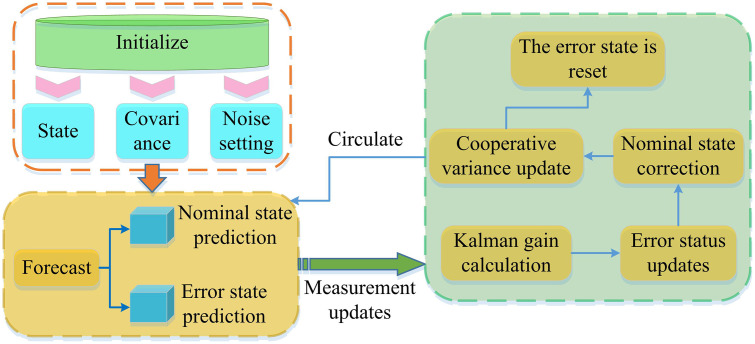
ESKF timetable.

Robots often face multiple challenges brought by complex dynamic environments in practical application scenarios such as autonomous driving and special operations. In practical applications, the state estimation method of a single sensor is limited by the characteristics of the sensor itself and difficult to adapt to complex and changing environments. Existing Multi-Sensor Fusion (MSF) strategies often use simple data concatenation or parallel fusion modes, which do not fully consider the spatio-temporal asynchrony and observation confidence differences of each sensor data, resulting in insufficient consistency of the fused data and inability to maximize the collaborative advantages of multiple sensors [19,20]. Therefore, in response to the problems of noise interference, data loss, and motion target interference faced by robot multi-sensor systems in complex dynamic scenes, a multi-sensor system state estimation algorithm based on SF is proposed. The algorithm mainly consists of SF and ESKF, and the operation of ESKF is shown in Fig 1.

As shown in Fig 1, the design of a multi-sensor state estimation method first ensures the authenticity of state prediction by introducing noise, extracts effective observation information from sensors, and calculates packet loss compensation observation values. After integrating the data, the state mean and covariance are dynamically adjusted to improve the estimation reliability [21,22]. To meet the actual scenario of robots moving in complex environments (such as changes in the position and posture of autonomous driving robots), noise is introduced to ensure the authenticity of state prediction, as shown in equation (1).


$$m_{n+1}=f\left(m_{n}\right)+\Gamma_{n}z_{n}$$ (1)

**Fig 2:**
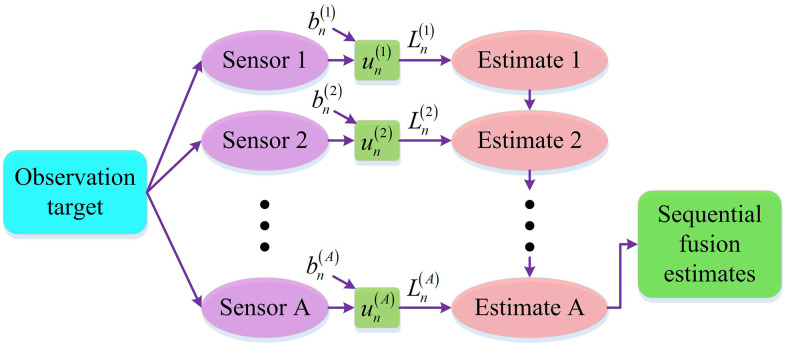
Sequential fusion structure diagram.

In equation (1), $m_{n+1}$ represents the system’s state at time n+1; f() means a nonlinear state transition function; $\Gamma_{n}$ means the covariance matrix of state transition noise; $z_{n}$ means the process noise. SF can achieve the optimal balance of performance while adapting to the practical engineering needs of multi-sensor systems. Therefore, studying the fusion of SF and ESKF for multi-sensor system state estimation, the structure of SF is shown in Fig 2.

As shown in Fig 2, the observation target passes through multiple sensors such as sensor 1 to sensor A. Each sensor combines the introduced noise to extract effective observation information and generate corresponding estimates, ultimately achieving SF estimation [23,24]. To further analyze the process of extracting effective observation information after introducing noise, the effective observation information of each sensor is extracted after introducing noise, as shown in equation (2).


$$u_{n}^{\left(a\right)}=w^{\left(a\right)}\left(m_{n}\right)+c_{n}^{\left(a\right)},a=1,2,\ldots ,A$$ (2)

In equation (2), $u_{n}^{\left(a\right)}$ means the observation value of the a th sensor at time n; $w^{\left(a\right)}\left(\right)$ means the nonlinear observation function of the a th sensor; $c_{n}^{\left(a\right)}$ stands for observation noise; A is the total number of sensors. Then, the packet loss compensation observation is calculated to mitigate the impact of missing data from a single sensor on the overall state estimation, ensuring data continuity and reducing estimation bias caused by packet loss, as shown in equation (3).


$$L_{n}^{\left(a\right)}=b_{n}^{\left(a\right)}u_{n}^{\left(a\right)}+\left(1-b_{n}^{\left(a\right)}\right)u_{n|n-1}^{\left(a\right)}$$ (3)

In equation (3), $L_{n}^{\left(a\right)}$ is the compensated observation value of the a th sensor at time n; $b_{n}^{\left(a\right)}$ is the packet loss compensation coefficient; $u_{n|n-1}^{\left(a\right)}$ is the observation value predicted based on the observation value at time n−1. The observation data, integrated with packet loss compensation, is shown in equation (4).


$$\left\{ \begin{array}{c} \prod_{a}\left(n+1\right)=\left\{L_{n+1}^{\left(1\right)},L_{n+1}^{\left(2\right)},\ldots ,L_{n+1}^{\left(a\right)}\right\}\\ \prod \left(n+1\right)=\left\{\prod_{A}\left(0\right),\prod_{A}\left(1\right),\ldots ,\prod_{A}\left(n+1\right)\right\} \end{array}\right.$$ (4)

In equation (4), $\prod_{a}\left(n+1\right)$ is the information set of the a th sensor at time n+1, which includes all the observation data received from the sensor. These data are obtained after packet loss compensation processing; ∏(n+1) is the joint information set of all sensors at time n+1, which includes the information set of all sensors and is used for state estimation. By using conditional probability density to determine the degree of impact of newly added sensor information on state estimation, the state mean and covariance can be dynamically adjusted to improve the reliability of the estimation, as denoted in equation (5).


$$h\left(m_{n+1}|\prod \left(n\right),\prod_{a}\left(n+1\right)\right)=N\left(m_{n+1};\overset{―}{m_{n+1}^{a}},W_{n+1|n+1}^{m^{a}}\right),n\geq 0$$ (5)

In equation (5), $h\left(m_{n+1}|\prod \left(n\right),\prod_{a}\left(n+1\right)\right)$ is the conditional probability density function of state $m_{n+1}$ under the condition of all measurement information ∏(n) at the current time n and the measurement information $\prod_{a}\left(n+1\right)$ of the a th sensor at time n+1; $N\left(m_{n+1};\overset{―}{m_{n+1}^{a}},W_{n+1|n+1}^{m^{a}}\right)$ means a Gaussian normal distribution with a mean of $\overset{―}{m_{n+1}^{a}}$ and a covariance matrix of $W_{n+1|n+1}^{m^{a}}$, which describes the state estimation without fusing the information from the a th sensor. Information is progressively fused from the first sensor to the A th sensor, ultimately yielding a globally optimal estimate as shown in equation (6).


$$\left\{ \begin{array}{c} {\overset{―}{m}}_{n+1}^{j}={\overset{―}{m}}_{n+1}^{A}\\ W_{n+1|n+1}^{m^{j}}=W_{n+1|n+1}^{m^{A}} \end{array}\right.$$ (6)

**Fig 3:**
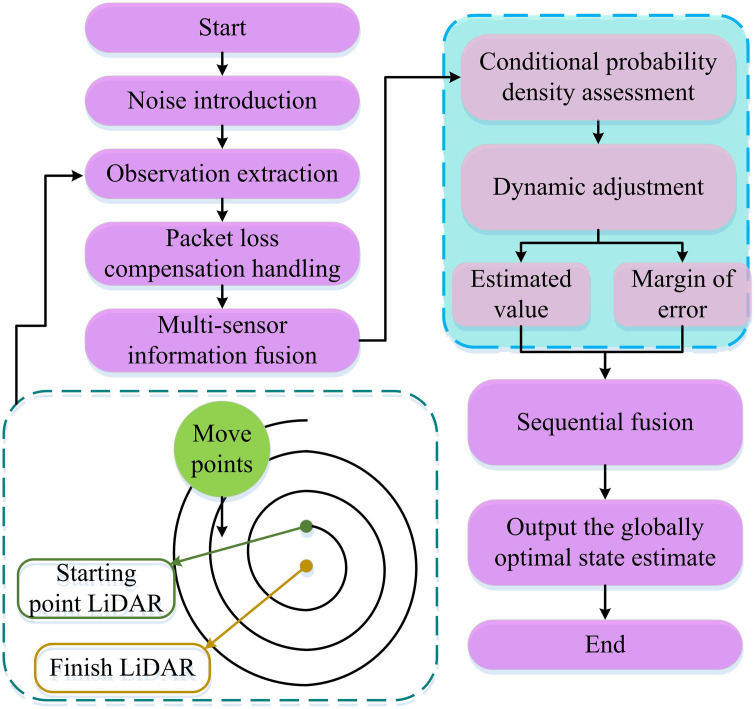
Flowchart for state estimation in multi-sensor systems.

In equation (6), ${\overset{―}{m}}_{n+1}^{j}$ represents the state estimation obtained by sequentially fusing all sensor information at time n+1; ${\overset{―}{m}}_{n+1}^{A}$ is the state estimation obtained by fusing information from the A th sensor at time n+1; ${\overset{―}{m}}_{n+1}^{N}$ means the covariance matrix of the state estimation error obtained by fusing all sensor information at time n+1; $W_{n+1|n+1}^{m^{A}}$ means the covariance matrix of the state estimation error after the fusion of information from the A th sensor at time n+1. Therefore, the process of state estimation for multi-sensor systems based on SF is shown in Fig 3.

As shown in Fig 3, the process involves introducing noise, extracting observations, compensating for packet loss, and fusing multi-sensor information. Then, the estimated values and error ranges are obtained through conditional probability density evaluation and dynamic adjustment. Subsequently, the information from each link is sequentially fused to output the globally optimal state estimation result [20].

### 2.2 Fusion of laser and visual detection data for motion target detection

The state estimation method for multi-sensor systems based on SF has achieved packet loss compensation and dynamic weight allocation for multi-source sensor data, laying the foundation for data integration and error suppression in complex dynamic environments. However, the interference of moving targets in dynamic scenes can directly affect the effectiveness of sensor observation data, and relying solely on data level fusion is difficult to completely eliminate the impact of such interference on the final state estimation results. Therefore, it is necessary to further combine the three-dimensional (3D) spatial perception capability of LiDAR with the target recognition advantages of visual sensors, and achieve precise detection and localization of moving targets through multimodal data fusion, thereby providing key technical support for optimizing state estimation accuracy and reducing errors caused by dynamic interference in the future [21,22]. First, the 3D point coordinates in the LiDAR Coordinate System (CS) are transformed into 3D point coordinates in the camera CS, achieving spatial coordinate alignment between the two sensors, as shown in equation (7).


$$\left[\begin{array}{c} {}*20cx_{1}\\ y_{1}\\ q_{1} \end{array}\right]=B\left[\begin{array}{c} {}*20cx_{2}\\ y_{2}\\ q_{2} \end{array}\right]+P$$ (7)

In equation (7), $x_{1}$, $y_{1}$, and $q_{1}$ are the coordinates of the point in the camera CS, respectively; $x_{2}$, $y_{2}$, and $q_{2}$ are the coordinates of the point in the LiDAR CS, respectively; B is a 3 × 3 rotation matrix used to describe the rotation relationship between two CSs and to rotate points from the LiDAR CS to the camera CS; P is a 3 × 1 translation vector used to describe the translation relationship between two CSs and to translate points from the LiDAR CS to the camera CS. Then, the LiDAR homogeneous coordinates are converted to camera homogeneous coordinates, as shown in equation (8).


$$\left[\begin{array}{c} {}*20cx_{1}\\ y_{1}\\ q_{1}\\ 1 \end{array}\right]=\left[\begin{array}{cc} {}*20cB & P\\ 0 & 1 \end{array}\right]\left[\begin{array}{c} {}*20cx_{2}\\ y_{2}\\ q_{2}\\ 1 \end{array}\right]$$ (8)

**Fig 4:**
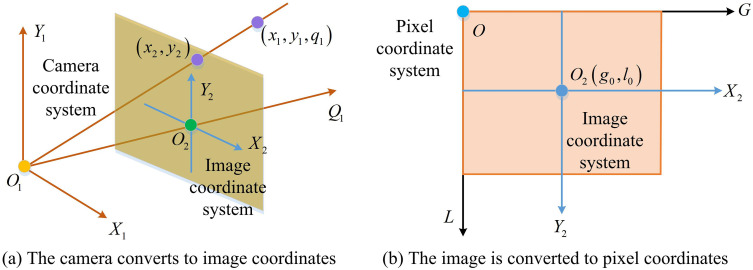
Coordinate system transformation schematic diagram.

In equation (8), $\left[\begin{array}{c} {}*20cx_{1}\\ y_{1}\\ q_{1}\\ 1 \end{array}\right]$ is the homogeneous coordinate of the point in the camera CS; $\left[\begin{array}{c} {}*20cx_{2}\\ y_{2}\\ q_{2}\\ 1 \end{array}\right]$ is the homogeneous coordinate of a point in the LiDAR CS; $\left[\begin{array}{cc} {}*20cB & P\\ 0 & 1 \end{array}\right]$ is a 4 × 4 transformation matrix that combines rotation and translation operations, used to convert points in the LiDAR CS to the camera CS. When detecting moving targets through LiDAR and camera fusion, only by converting the points in the LiDAR CS to the camera CS can the point cloud data and image data be matched for unified processing, achieve data fusion, and accurately detect and recognize targets. The principle of CS transformation is shown in Fig 4.

Fig 4(a) shows the transformation from camera CS to image CS, where light rays are projected onto the image plane to form image coordinate points, etc; Fig 4(b) shows the conversion process from the image CS to the pixel CS, clearly presenting the transition from the camera to the image and then to the pixel CS [23]. The 3D coordinates in the camera CS are projected onto the two-dimensional (2D) image plane to obtain the image plane coordinates, as shown in equation (9).


$$\left\{ \begin{array}{c} {x^{\prime }}_{o}=J\frac{x_{1}}{q_{1}}\\ {y^{\prime }}_{o}=J\frac{y_{1}}{q_{1}} \end{array}\right.$$ (9)

In equation (9), $x_{o}^{\prime }$ and $y_{o}^{\prime }$ are the coordinates of 2D points on the image plane, respectively; J is the focal length of the camera. The homogeneous CS of the camera’s 3D coordinates is scaled to output 2D image plane coordinates. This provides standardized 2D image plane coordinates for subsequent pixel coordinate transformations, ensuring projection accuracy across different cameras (or different calibration states of the same camera). This reduces detection errors caused by device variations, as shown in equation (10).


$$q_{1}\left[\begin{array}{c} {}*20c{x^{\prime }}_{o}\\ {y^{\prime }}_{o}\\ 1 \end{array}\right]=\left[\begin{array}{cccc} {}*20cJ & 0 & 0 & 0\\ 0 & J & 0 & 0\\ 0 & 0 & 1 & 0 \end{array}\right]\left[\begin{array}{c} {}*20cx_{1}\\ y_{1}\\ q_{1}\\ 1 \end{array}\right]$$ (10)

In equation (10), $\left[\begin{array}{cccc} {}*20cJ & 0 & 0 & 0\\ 0 & J & 0 & 0\\ 0 & 0 & 1 & 0 \end{array}\right]$ is a 3 × 4 projection matrix that scales the coordinates in homogeneous coordinates according to the focal length J, while keeping the $q_{1}$ coordinates unchanged. Correcting the system deviation from image plane coordinates to pixel coordinates yields the final pixel coordinates of the moving target within the image, as shown in equation (11).


$$\left\{ \begin{array}{c} g=\frac{{x^{\prime }}_{o}}{dx}+g_{0}\\ l=\frac{{y^{\prime }}_{o}}{dy}+l_{0} \end{array}\right.$$ (11)

In equation (11), $g_{0}$ and $l_{0}$ are the offset of pixel coordinates, which may be due to camera installation pose (such as image origin offset caused by camera optical axis not overlapping with laser CS), calibration error compensation, etc., used to correct system deviation during the conversion from physical coordinates to pixel coordinates, ensuring the accuracy of target localization on the image after laser and visual data fusion; $g_{0}$ and l are the pixel coordinates of the target on the image plane, corresponding to the horizontal and vertical pixel positions of the moving target in the visual detection image. They are the basic data output of visual detection and used to locate the location of the target in the image. By integrating the above formulas, the conversion from the 3D physical coordinates of the camera to the 2D pixel coordinates of the image can be achieved uniformly, as shown in equation (12).


$$\left[\begin{array}{c} {}*20cg\\ l\\ 1 \end{array}\right]=\left[\begin{array}{ccc} {}*20c\frac{\text{1}}{dx} & 0 & g_{0}\\ 0 & \frac{\text{1}}{dy} & l_{0}\\ 0 & 0 & 1 \end{array}\right]\left[\begin{array}{c} {}*20c{x^{\prime }}_{o}\\ {y^{\prime }}_{o}\\ 1 \end{array}\right]$$ (12)

**Fig 5:**
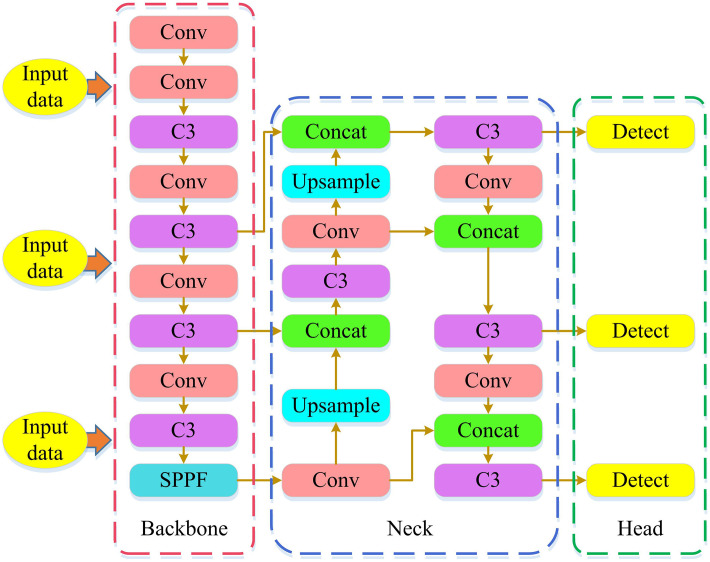
YOLOv5s architecture diagram.

In equation (12), $\left[\begin{array}{ccc} {}*20c\frac{\text{1}}{dx} & 0 & g_{0}\\ 0 & \frac{\text{1}}{dy} & l_{0}\\ 0 & 0 & 1 \end{array}\right]$ is a 3 × 3 transformation matrix that combines scaling and translation operations, converting physical coordinates in the camera CS to pixel coordinates in the image CS; $\left[\begin{array}{c} {}*20cg\\ l\\ 1 \end{array}\right]$ is the pixel coordinate in the image CS. In dynamic scenes, moving targets such as pedestrians and other mobile devices can interfere with the validity of sensor observation data, leading to bias in state estimation. YOLOv5s, with its lightweight characteristics, can efficiently identify moving targets within the coverage area of LiDAR and visual sensors while ensuring a certain detection accuracy. It can accurately locate the pixel coordinates of targets in the image (by combining with CS conversion to achieve the correspondence between laser point cloud and visual image data), providing a basis for eliminating the interference of moving targets on state estimation in the future, and reducing false positives and false negatives caused by target motion blur and occlusion [25]. The structure of YOLOv5s algorithm is denoted in Fig 5.

As shown in Fig 5, starting from the input data, the Backbone module first extracts features, then the Neck module performs feature fusion and upsampling, and finally the Head’s Detect module completes object detection. By utilizing YOLOv5s to balance the contributions of the three loss functions, the overall performance of motion object detection is enhanced, as shown in equation (13).


$$F=\alpha F_{1}+\beta F_{2}+\lambda F_{3}$$ (13)

In equation (13), F is the total loss function, which is the weighted sum of different loss terms; α, β, and λ are different weight parameters used to balance the contributions of different loss items in the total loss; $F_{1}$ stands for classification loss, which is used to predict the difference between a category and the true category; $F_{2}$ is the target loss used to measure the accuracy of predicting the presence or absence of a target; $F_{3}$ is the localization loss used to measure the difference between the predicted bounding box and the true bounding box. The false positive and false negative caused by target motion blur and occlusion in dynamic scenes can be better distinguished by binary cross entropy loss to help the model distinguish the presence or absence of targets, further reducing the false negative rate. The calculation formula is shown in equation (14).


$$F_{BCE}=-\frac{\text{1}}{K}\sum {\mathrm{\backslash nolimits}}_{k}\left[e\ln r+\left(1-e\right)\ln\left(1-r\right)\right]$$ (14)

In equation (14), $F_{BCE}$ is the binary cross entropy loss, which is a loss function used for classification problems and is used in object detection to evaluate the difference between the predicted class probability of the model and the true class; K is the number of samples, which refers to the number of images or frames used during the training process; e is the true label, which means whether the target exists or not; r is the probability of predicting the existence of the target. In dynamic scenes, moving targets may change in shape due to changes in posture. By calculating, both position and shape deviations can be corrected simultaneously, improving the fit between the bounding box and the real target, as shown in equation (15).


$$F_{3}=1-F_{IoU}+\frac{S^{2}\left(v^{\prime },v\right)}{d^{2}}+\kappa \varphi$$ (15)

**Fig 6:**
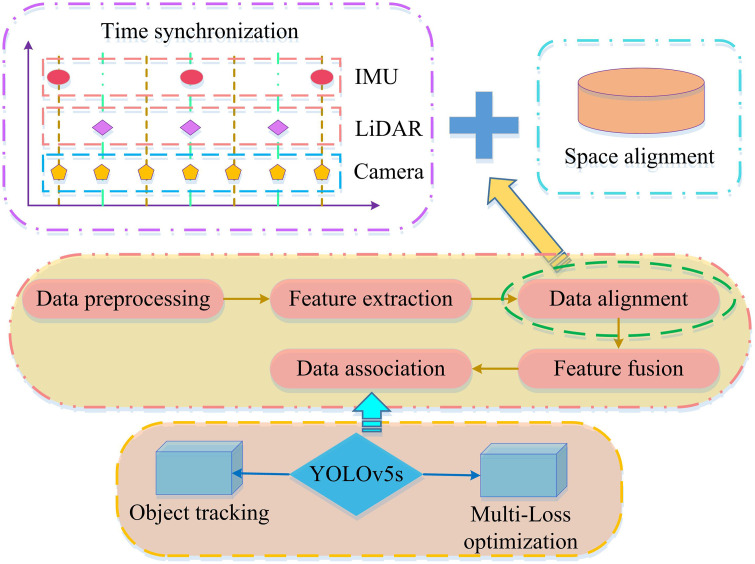
Multi-feature fusion diagram.

In equation (15), $F_{IoU}$ is the intersection to union ratio, which measures the degree of overlap between two bounding boxes. In object detection, the accuracy of predicting bounding boxes is evaluated by calculating the intersection to union ratio of two bounding boxes; $S^{2}\left(v^{\prime },v\right)$ is the square of the distance between the predicted bounding box $v^{\prime }$ and the real bounding box v; d is the diagonal length of the smallest bounding box (the smallest rectangle that covers both bounding boxes) where the predicted bounding box and the real bounding box are located; φ and κ are coefficients that limit the shape (aspect ratio) of the bounding box. Then, multiple data from laser and visual inspection are fused, as shown in Fig 6.

As shown in Fig 6, the sensor data is first time-synchronized to ensure temporal consistency, followed by spatial alignment for analysis within a unified CS. Data alignment and feature fusion are then performed through preprocessing and feature extraction to obtain richer feature representations. Finally, object tracking is conducted using the YOLOv5s algorithm, with tracking accuracy enhanced through multi-loss optimization.

### 2.3 Construction of sequential fusion odometer based on error-state Kalman filter

**Fig 7:**
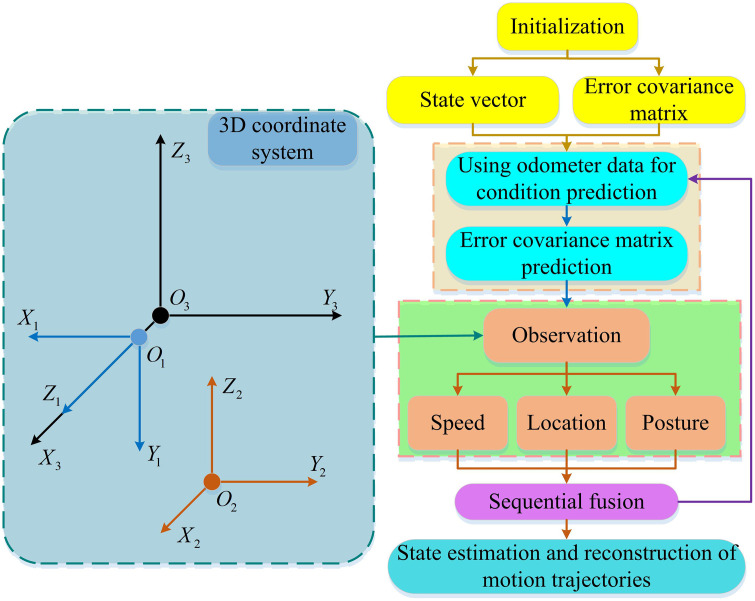
Flowchart of the sequential fusion odometer based on ESKF.

As the core part of robot positioning and navigation, the accuracy of odometer directly determines the reliability of equipment motion control and path planning. There are obvious limitations in traditional single sensor odometry methods: wheeled odometry is prone to cumulative errors due to ground slippage, inertial measurement units are affected by zero bias drift and have poor short-term accuracy, and visual odometry is prone to failure in scenarios with changes in lighting or missing features. MSF can greatly improve the performance of odometry. By aligning the spatial coordinates of LiDAR and visual sensors, performing homogeneous coordinate transformation, and balancing the optimization of classification loss, target loss, and positioning loss using YOLOv5s algorithm, accurate detection of moving targets in dynamic scenes and multi-modal data feature fusion can be further achieved. This effectively reduces the problem of false detection and missed detection caused by blurry and occluded moving targets, and eliminates key interference sources for subsequent system state estimation. On this basis, to further improve the accuracy of robot motion positioning and trajectory reconstruction, the error correction capability of ESKF is deeply combined with the advantage of SF of multi-sensor information integration to construct an SF odometer adapted to complex dynamic environments, as shown in Fig 7.

As shown in Fig 7, the raw data from the LiDAR, vision sensor, and inertial measurement unit were first acquired. Standardized fused data was output through time synchronization (calibrating the time of multiple sensors based on high-frequency sampling of the IMU) and spatial coordinate transformation (transforming the LiDAR 3D point cloud to the camera CS through rotation and translation, and then projecting it onto the image pixel coordinates). Then, YOLOv5s was used to improve the detection accuracy. Next, the pixel coordinates were reverse-mapped to the LiDAR 3D coordinates to generate a dynamic target spatial mask. Then, the preprocessed data was retrieved through the SF framework, and the data gaps were filled by the packet loss compensation formula. The sensor data weights were adjusted in combination with the dynamic target mask (the weight of dynamic areas was reduced, and the weight of static areas was maintained). At the same time, ESKF was embedded throughout the entire process. It first introduced noise through the state transition formula to ensure the authenticity of the prediction, and then dynamically adjusted the state mean and covariance based on the conditional probability density to correct sensor noise and IMU drift errors. Finally, it integrated all sensor information and error correction results, built an SF odometry, and output state parameters such as robot position, attitude, and speed, forming a complete closed loop of interference elimination, data fusion, and error suppression.

## 3 Results

### 3.1 Performance verification and comparative analysis of error-state Kalman filter

To ensure the pertinence and effectiveness of the experiment, the study selected EKF-YOLOv5s, YOLOv5s, ESKF-LiDAR, and Transformer-LiDAR as baseline algorithms. These algorithms have certain advanced representation in the fields of multi-sensor state estimation and robot positioning. EKF is a traditional classic filtering algorithm in the field of multi-sensor fusion. EKF-YOLOv5s adopts a filtering-target detection fusion framework. Only the filtering module is different. As a classic framework, it can independently verify the suppression effect of ESKF on nonlinear errors. YOLOv5s is a mainstream model in the field of lightweight target detection. It only retains the target detection function and has no filter module. It can quantify the advantages of the filter-detection fusion architecture compared to a single detection technology. As a benchmark for lightweight target detection, it has become a basic detection model in resource-constrained scenarios with small parameters, fast reasoning, and balanced accuracy, and represents the advanced direction of lightweight visual detection. LiDAR is a single sensor, which contrasts with multi-sensor fusion and can verify the improvement of state estimation by the complementarity of multi-source data. At the same time, ESKF is used to eliminate interference from differences in filtering algorithms. Transformer represents an emerging technology. Comparing it with traditional filter fusion (ESKF-YOLOv5s) can quantify the difference in performance between the two technologies, verify the value of research on improving traditional filtering technology, and then highlight its real-time advantages over emerging feature fusion technology.

To verify the accuracy of the MSF state estimation algorithm based on ESKF-YOLOv5s, data were randomly selected from the KITTI dataset, and EKF-YOLOv5s, YOLOv5s, Transformer-LiDAR, and ESKF-LiDAR state estimation algorithms were selected for comparative experiments. The estimated values of the algorithms were recorded separately, and the error between the estimated values and the true values was calculated, as shown in Table 1.

**Table 1:** Error analysis of estimation algorithms.

Algorithm	Estimated value		Actual value		Error
	x	y	x	y	
ESKF-YOLOv5s	18.23	6.78	18.01	6.55	0.36
EKF-YOLOv5s	18.56	6.99	18.01	6.55	0.49
YOLOv5s	19.12	7.34	18.01	6.55	0.82
Transformer-LiDAR	18.34	6.89	18.01	6.55	0.41
ESKF-LiDAR	18.45	7.01	18.01	6.55	0.48

**Fig 8:**
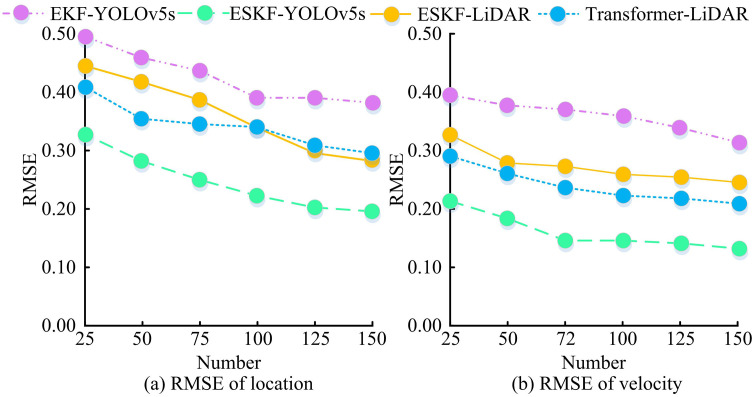
RMSE curve for estimating the position and velocity of the algorithm.

According to Table 1, the estimated value x of ESKF-YOLOv5s algorithm was 18.23, y was 6.78, and the error was only 0.36, which was the smallest among all compared algorithms; The estimated values of x and y by the Transformer-LiDAR algorithm were 18.34 and 6.89, respectively, with an error of 0.41, second only to ESKF-YOLOv5s; The ESKF-LiDAR algorithm estimated x to be 18.45, y to be 7.01, with an error of 0.48; The EKF-YOLOv5s algorithm estimated x to be 18.56 and y to be 6.99, with an error of 0.49. These two algorithms had similar errors and were slightly higher than Transformer-LiDAR; The YOLOv5s algorithm estimated x to be 19.12 and y to be 7.34, with an error of 0.82, which was the largest error among all compared algorithms. Comparison showed that the ESKF-YOLOv5s algorithm had the smallest error and significantly better accuracy than the comparison algorithm. To verify the stability of the MSF state estimation algorithm based on ESKF-YOLOv5s, 150 sets of data were randomly selected from the KITTI dataset. The estimation algorithms of ESKF-YOLOv5s, EKF-YOLOv5s, Transformer-LiDAR, and ESKF-LiDAR were set up for experiments, and the Root Mean Square Error (RMSE) and velocity RMSE of the algorithms were calculated at different data volumes, as shown in Fig 8.

**Fig 9:**
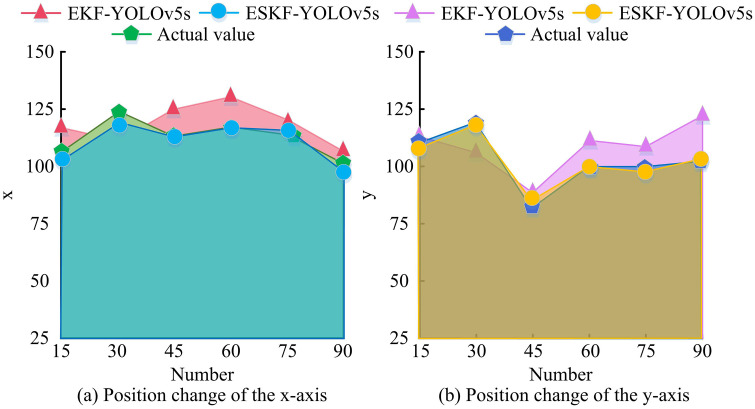
Comparison of estimated trajectories from estimation algorithms and actual trajectories.

In Fig 8(a), as the amount of data increased, the position RMSE curve of the ESKF-YOLOv5s algorithm continued to decrease, from 0.33 to 0.20; The initial RMSE of the EKF-YOLOv5s algorithm was 0.50, which decreased to 0.38; The position RMSE of ESKF-LiDAR algorithm decreased from 0.44 to 0.28; The Transformer-LiDAR algorithm was reduced from 0.41 to 0.29. In Fig 8(b), as the data volume increased, the RMSE curve of the velocity of ESKF-YOLOv5s decreased from 0.21 to 0.13; EKF-YOLOv5s decreased from 0.39 to 0.31; ESKF-LiDAR decreased from 0.33 to 0.24; Transformer-LiDAR reduced from 0.29 to 0.21. The results showed that the RMSE of the ESKF-YOLOv5s algorithm for position and velocity estimation gradually decreased with increasing data volume. Comparison showed that the ESKF-YOLOv5s algorithm outperformed the comparative algorithm in terms of accuracy and stability of position and velocity estimation, with significant advantages. To further validate the accuracy of the MSF state estimation algorithm based on ESKF-YOLOv5s, 90 sets of data were randomly selected from the KITTI dataset and the experiment was repeated 10 times. The estimation trajectories of the ESKF-YOLOv5s and EKF-YOLOv5s estimation algorithms were recorded, as shown in Fig 9.

**Fig 10:**
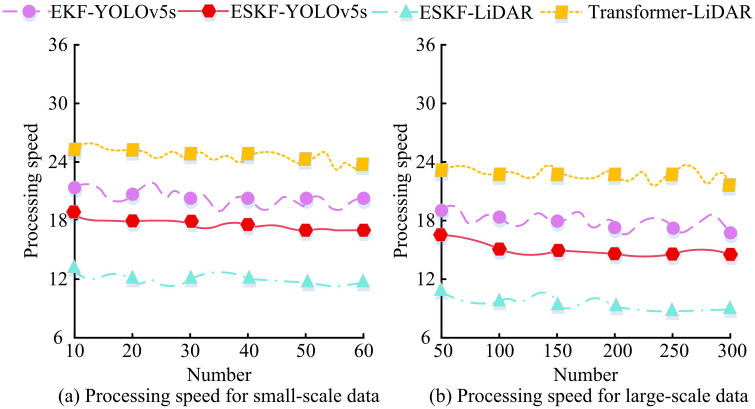
Processing speed of estimation algorithms across different data sizes.

Fig 9(a) shows the change in x-axis position, where the ESKF-YOLOv5s (blue) trajectory closely matched the true value (green). At data points 30 and 60, the estimated value had minimal deviation from the true value; The EKF-YOLOv5s (red) trajectory deviated significantly, with large deviations at data points 15 and 75. Fig 9(b) shows the change in y-axis position, where the ESKF-YOLOv5s (yellow) trajectory closely matched the true value (blue) and almost overlapped at data points 45 and 60; The EKF-YOLOv5s (purple) trajectory fluctuated greatly and deviated significantly, with prominent errors at data points 30 and 90. Comparison showed that the ESKF-YOLOv5s algorithm estimated trajectories closer to real trajectories in the x and y directions, indicating better accuracy. In addition, to verify the computational efficiency of the MSF state estimation algorithm based on ESKF-YOLOv5s, 60 sets of (small-scale) and 300 sets of (large-scale) data were randomly selected from the KITTI dataset, and the processing speed (ms/frame) of ESKF-YOLOv5s, EKF-YOLOv5s, Transformer-LiDAR, and ESKF-LiDAR estimation methods for data of different scales were recorded, as shown in Fig 10.

Fig 10(a) shows small-scale data. The processing speed of ESKF-LiDAR algorithm remained stable at 12ms/frame, with the highest efficiency; The processing speed of ESKF-YOLOv5s algorithm remained at 18ms/frame, with small fluctuations and overall stability; The processing speed of EKF-YOLOv5s algorithm was 20ms/frame; The processing speed of Transformer-LiDAR was 25ms/frame, which was relatively slow. Fig 10(b) shows large-scale data. The ESKF-LiDAR algorithm maintained a relatively efficient processing speed of 9ms/frame; The processing speed of ESKF-YOLOv5s algorithm was 16ms/frame; The processing speeds of EKF-YOLOv5s algorithm and Transformer-LiDAR algorithm were 18ms/frame and 24ms/frame, respectively. Overall comparison showed that regardless of whether it was small-scale or large-scale data, ESKF-LiDAR had the best processing speed, followed by ESKF-YOLOv5s, showing differences in computational efficiency among different algorithms in MSF state estimation. This also indicated that due to the influence of multi-sensor data, the processing ability of ESKF-YOLOv5s algorithm was lower than that of ESKF-LiDAR algorithm with a single sensor. However, ESKF-YOLOv5s algorithm had more advantages in multi-data processing speed, and ESKF-YOLOv5s algorithm provided reference for the efficiency selection of algorithm practical applications.

To verify the generalization ability of the MSF state estimation algorithm based on ESKF-YOLOv5s, the ESKF-YOLOv5s, ESKF-YOLOv8n, ESKF-YOLOv7-tiny, EKF-YOLOv5s, and YOLOv5s algorithms were selected for experiments. 240 groups of samples were selected from the KITTI data set (conventional urban roads) and the NuScenes data set (complex cities) to test and record the parameter amount (M), inference time (ms/frame), energy consumption (W), and mean average precision (mAP) of the algorithm. The mAP index adopts the evaluation standard with an IoU threshold of 0.5 (mAP@0.5), as shown in Table 2.

**Table 2:** Comparison of algorithm metrics.

Algorithm	KITTI				NuScenes			
	Parameter quantity	Time	Energy	mAP@0.5	Parameter quantity	Time	Energy	mAP@0.5
ESKF-YOLOv5s	7.2	18.5	85.3	0.89	7.2	20.1	88.7	0.85
ESKF-YOLOv8n	6.1	16.2	81.5	0.86	6.1	17.9	84.3	0.81
ESKF-YOLOv7-tiny	8.9	22.7	90.1	0.84	8.9	24.5	93.5	0.79
EKF-YOLOv5s	7.2	20.3	87.6	0.82	7.2	22.5	90.2	0.76
YOLOv5s	7.2	17.8	83.2	0.78	7.2	19.6	85.8	0.71

According to Table 2, ESKF-YOLOv5s had the same parameter amount as EKF-YOLOv5s and YOLOv5s (7.2M), which was slightly higher than ESKF-YOLOv8n (6.1M) and lower than ESKF-YOLOv7-tiny (8.9M). On the KITTI data set, the inference time of ESKF-YOLOv5s (18.5ms/frame) was higher than ESKF-YOLOv8n (16.2ms/frame) and YOLOv5s (17.8ms/frame), and was lower than ESKF-YOLOv7-tiny (22.7ms/frame) and EKF-YOLOv5s (20.3ms/frame). On the NuScenes dataset, their inference time (20.1ms/frame) still maintained a relative advantage, only higher than ESKF-YOLOv8n (17.9ms/frame), and was lower than the other three algorithms. The energy consumption of ESKF-YOLOv5s was at a medium level on both data sets; ESKF-YOLOv5s had the highest mAP@0.5 on both data sets, the KITTI data set (0.89) and the NuScenes data set (0.85). The data shows that the ESKF-YOLOv5s algorithm has a balanced performance in parameter quantity, inference time, energy consumption, and detection accuracy, and shows excellent performance on different scene data sets, with outstanding generalization ability.

### 3.2 Practical application scenarios and scenario adaptability analysis

To verify the effectiveness of the MSF state estimation algorithm based on ESKF-YOLOv5s in practical application scenarios, EKF-YOLOv5s, YOLOv5s, Transformer-LiDAR, and ESKF-LiDAR state estimation systems were selected for comparative experiments. The RMSE, Mean Squared Error (MSE), and Structural Similarity Index Measure (SSIM) of the algorithm were tested in dynamic and static scenarios, respectively, as shown in Table 3.

**Table 3:** Analysis of the estimation system’s performance in real-world application scenarios.

System	Dynamic scene			Static scene		
	RMSE	MSE	SSIM	RMSE	MSE	SSIM
ESKF-YOLOv5s	0.23	0.15	0.92	0.12	0.08	0.95
EKF-YOLOv5s	0.25	0.18	0.89	0.15	0.11	0.90
YOLOv5s	0.35	0.32	0.85	0.22	0.18	0.82
Transformer-LiDAR	0.29	0.23	0.87	0.14	0.09	0.93
ESKF-LiDAR	0.27	0.21	0.89	0.13	0.09	0.94

According to Table 3, the RMSE (0.23) and MSE (0.15) of ESKF-YOLOv5s in dynamic scenes were lower than ESKF-LiDAR (0.27, 0.21), YOLOv5s (0.35, 0.32), Transformer-LiDAR (0.29, 0.23), EKF-YOLOv5s (0.25, 0.18). The SSIM (0.92) of ESKF-YOLOv5s was higher than other algorithms; In static scenarios, the RMSE (0.12) and MSE (0.08) of ESKF-YOLOv5s were optimal, and the SSIM (0.95) was also higher than EKF-YOLOv5s (0.90), YOLOv5s (0.82), Transformer-LiDAR (0.93), and ESKF-LiDAR (0.94). The comparison results showed that ESKF-YOLOv5s performed better in state estimation than the comparison algorithms in multiple scenarios, especially in dynamic scenarios where accuracy, stability, and map quality were significantly improved. At the same time, it also had significant performance in static scenes. To further verify the reliability of the MSF state estimation algorithm based on ESKF-YOLOv5s, a two-tailed t test was used, and the significance level α = 0.05 was set. Taking ESKF-YOLOv5s as the experimental group, EKF-YOLOv5s, YOLOv5s, Transformer-LiDAR, and ESKF-LiDAR as the control groups respectively, it tested whether the difference in position RMSE and MSE between the two groups of algorithms in dynamic and static scenes is statistically significant (*p* < 0.05 means the difference is significant), as shown in Table 4.

**Table 4:** Significance test results.

System	Dynamic scene		Static scene		Conclusion
	RMSE(*p*-value)	MSE(*p*-value)	RMSE(*p*-value)	MSE(*p*-value)	
EKF-YOLOv5s	0.032	0.028	0.035	0.025	The difference is significant
YOLOv5s	0.008	0.006	0.009	0.007	The difference is significant
Transformer-LiDAR	0.038	0.031	0.042	0.036	The difference is significant
ESKF-LiDAR	0.026	0.021	0.039	0.033	The difference is significant

**Fig 11:**
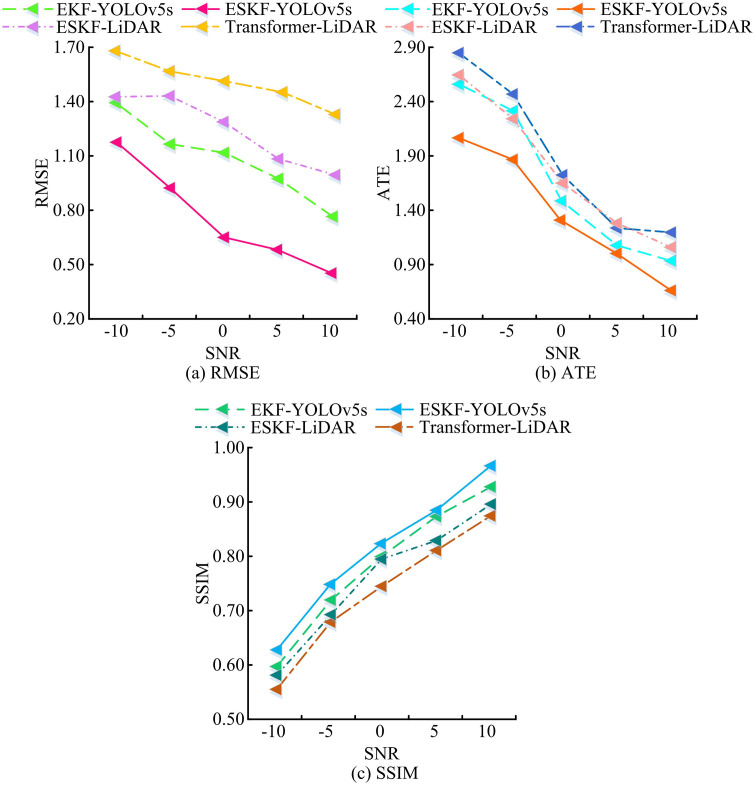
Variation in RMSE and ATE of estimation systems across different noise environments.

According to Table 4, in dynamic and static scenarios, the *p* values of RMSE and MSE comparing ESKF-YOLOv5s with EKF-YOLOv5s, YOLOv5s, Transformer-LiDAR, and ESKF-LiDAR were all less than 0.05. The data shows that the accuracy advantage of ESKF-YOLOv5s has significant statistical significance. To verify the anti-interference ability of the MSF state estimation algorithm based on ESKF-YOLOv5s, experiments were conducted in environments with Signal-to-Noise Ratios (SNR) of -10dB, -5dB, 0dB, 5dB, and 10dB. The RMSE, Absolute Trajectory Error (ATE), and SSIM of the estimation algorithms based on ESKF-YOLOv5s, EKF-YOLOv5s, Transformer-LiDAR, and ESKF-LiDAR were measured, as shown in Fig 11.

**Fig 12:**
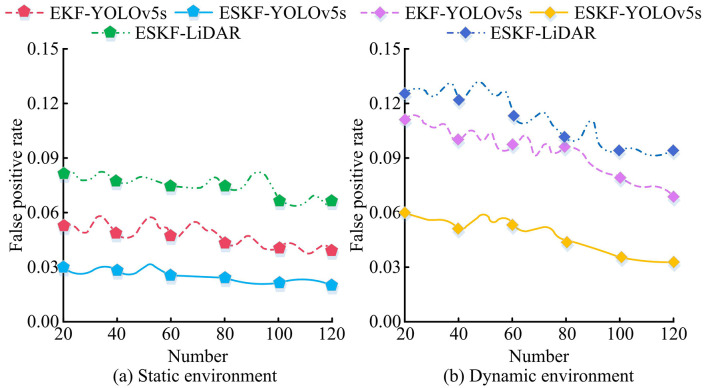
Estimated false positive rate of the system under different dynamic target interferences.

In Fig 11(a), as the SNR increased from -10dB to 10dB, the RMSE value of ESKF-YOLOv5s decreased significantly from 1.17 to 0.45; The RMSE curve of EKF-YOLOv5s gradually decreased from 1.39 to 0.77; The RMSE of Transformer-LiDAR decreased from 1.68 to 1.33; The RMSE of ESKF-LiDAR decreased from 1.43 to 0.99. In Fig 11(b), as the SNR changed, the ATE value of ESKF-YOLOv5s decreased from 2.07 to 0.66; EKF-YOLOv5s reduced from 2.57 to 0.93; The ATE of Transformer-LiDAR decreased from 2.85 to 1.21; The ATE curve of ESKF-LiDAR continued to decrease from 2.65 to 1.06. In Fig 11(c), as the SNR increased, the SSIM of all algorithms increased, with the ESKF-YOLOv5s algorithm consistently having the highest SSIM. In summary, under different noise environments, the optimization amplitude of RMSE, ATE, and SSIM of ESKF-YOLOv5s with SNR variation was better than that of the comparative algorithm, and its anti-interference performance was more outstanding. To further verify the anti-interference ability of the MSF state estimation algorithm based on ESKF-YOLOv5s, estimation algorithms based on ESKF-YOLOv5s, EKF-YOLOv5s, and ESKF-LiDAR were selected for experiments, and the false positive rates of the estimation algorithms in static environment and dynamic target interference were recorded, as shown in Fig 12.

**Fig 13:**
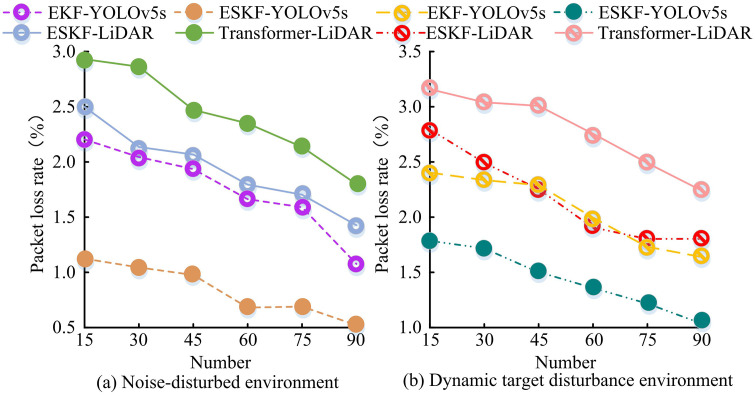
Estimated packet loss rate of the system in different environments.

In Fig 12(a), as the data volume changed, the false positive rate of ESKF-YOLOv5s remained stable in the range of [0.020, 0.030] with minimal fluctuations. At data points 20, 40, and 60, the false positive rate was close to 0.030; The false positive rate of EKF-YOLOv5s fluctuated within the range of [0.039, 0.053]; The false positive rate of ESKF-LiDAR remained at [0.065, 0.081], with higher overall false positive rates and slight fluctuations within data points 80–100. In Fig 12(b), as the data volume increased, the false positive rate of ESKF-YOLOv5s showed a decreasing trend, from 0.060 to 0.033, with a significant decrease and small fluctuations, always lower than other algorithms; The false positive rate of EKF-YOLOv5s fluctuated between [0.069, 0.111], although there was a slight decrease, the overall false positive rate was high; The false positive rate of ESKF-LiDAR fluctuated between [0.094, 0.126], with data point 20 being 0.126. The downward trend was not significant, and the false positive rate was also higher than that of ESKF-YOLOv5s. The experimental results showed that ESKF-YOLOv5s had better false positive rate control, significantly better anti-interference ability and state estimation robustness. In addition, to verify the stability of the MSF state estimation algorithm based on ESKF-YOLOv5s, 15 repeated tests were conducted in noisy and dynamic target interference environments. The packet loss rates of the estimation systems based on ESKF-YOLOv5s, EKF-YOLOv5s, Transformer-LiDAR, and ESKF-LiDAR were detected and recorded as the computational complexity increased, as shown in Fig 13.

In Fig 13(a), as the computational load increased, the packet loss rate of ESKF-YOLOv5s decreased from 1.12% to 0.53%, with a clear downward trend and the lowest value; ESKF-LiDAR decreased from 2.50% to 1.42%; Transformer-LiDAR decreased from 2.92% to 1.80%; EKF-YOLOv5s decreased from 2.20% to 1.07%, and the packet loss rates of the latter three were higher than ESKF-YOLOv5s. In Fig 13(b), as the computational load increased, the packet loss rate of ESKF-YOLOv5s decreased from 1.78% to 1.07%, with a continuous decrease and small fluctuations; ESKF-LiDAR decreased from 2.78% to 1.77%; Transformer-LiDAR decreased from 3.17% to 2.25%; EKF-YOLOv5s decreased from 2.40% to 1.65%. In summary, under both interference environments, the packet loss rate of ESKF-YOLOv5s decreased with increasing computational load, and its value was lower than other algorithms. It performed better in stability testing, and the stability of MSF state estimation algorithm was more prominent. To verify the stability of the MSF state estimation algorithm based on ESKF-YOLOv5s, the experiment set up the robot to conduct 15 repeated experiments in different terrain environments A1, A2, A3, A4, and A5, recording the corresponding estimated and actual positions, as shown in Table 5.

**Table 5:** Estimated position comparison of the system across different terrain environments.

Condition	Actual value		Estimated value		Error
	x	y	x	y	
A1	10.00	5.00	9.98	5.02	0.03
A2	20.00	12.00	19.95	12.06	0.08
A3	30.00	8.00	29.90	8.12	0.16
A4	15.00	20.00	15.05	19.92	0.10
A5	25.00	15.00	25.10	15.08	0.13

According to Table 5, among the five terrain environments A1-A5, the error in A1 environment was the smallest (0.03), the error in A3 environment was the largest (0.16), and the errors in other environments were in the range of [0.08, 0.13]. Overall, the environmental errors are at a relatively low level, indicating that the algorithm has controllable position estimation bias in diverse terrains, demonstrates good stability, and can adapt to state estimation in different terrains.

## 4 Discussion

Experimental verification showed that the performance of ESKF-YOLOv5s was significantly better than the compared algorithms. From the perspective of state estimation methods, the measurement sequence fusion method proposed by W. Liu et al focused on situations where the probability distribution of noise was unknown. With the unique region family construction and measurement value temporal fusion strategy, it achieved high accuracy and good time performance in turbofan engine control simulation. However, this method mainly addressed noise issues and lacked effective mechanisms to deal with dynamic target interference in dynamic scenes. It is not as adaptable to complex scenes as the ESKF-YOLOv5s algorithm, which uses ESKF to fuse multi-sensor data and combines YOLOv5s to detect moving targets, and can better handle multi-source data and interference factors in complex environments, ensuring state estimation accuracy [26]. D. V. Nam et al. developed an adaptive robust state estimation scheme for autonomous mobile robots based on a framework of second-class fuzzy inference systems and factor graph optimization. The experiment confirmed its accuracy and robustness, but the framework was relatively complex in terms of sensor fusion strategy and highly dependent on specific mobile robot hardware platforms. In contrast, the ESKF-YOLOv5s algorithm has a more flexible and universal way of integrating LiDAR and visual sensor data. With the powerful object detection capability of YOLOv5s, it can stably improve the reliability of state estimation in different scenarios, and has better computational efficiency and universality performance [27]. Y. Luo et al. conducted research on secure fuzzy network state estimation for 2D systems, using fuzzy theory and various analysis techniques to derive error dynamics and ensure safety. However, this method is limited to the specific structure and fuzzy theory application of 2D systems, and lacks universality in MSF and state estimation error processing. The ESKF-YOLOv5s algorithm, relying on Kalman filtering and object detection technology, can be widely applied to various multi-sensor systems, and has more advantages in state estimation performance in complex scenarios [28]. The ESKF-YOLOv5s algorithm focuses on state estimation in multi-sensor systems, and its object detection serves the overall state estimation needs. It can further optimize detection results using multi-sensor data and has stronger collaborative processing capabilities for object detection and state estimation in complex multi-source data fusion scenarios.

In summary, ESKF-YOLOv5s stands out in the field of multi-sensor system state estimation due to its SF strategy for multi-sensor data and its ability to detect moving targets. It demonstrates significant performance advantages in state estimation tasks in complex dynamic scenes by integrating the SF framework with YOLOv5s. Applying it to localization, mapping, and dynamic interference processing in scenarios such as robot autonomous driving and special operations is feasible and effective. Compared with other algorithms, ESKF-YOLOv5s is driving multi-sensor state estimation technology towards more accurate and intelligent directions in terms of applicable scenarios, problem-solving entry points, and technical implementation paths. Future research can further explore the integration of the advantages of these algorithms to cope with more complex and diverse practical application scenarios.

## 5 Conclusion

An SF-based multi-sensor system state estimation algorithm was proposed to address the problems of noise interference, sensor data loss, and motion target interference faced by robot multi-sensor systems in dynamic scenes. The YOLOv5s object detection model was then fused to construct an SF odometer adapted to complex dynamic environments, effectively improving the accuracy, stability, and anti-interference ability of robot state estimation. In the accuracy test of multi-sensor state estimation, data was selected from the KITTI dataset and compared with multiple algorithms. The ESKF-YOLOv5 algorithm’s estimated value (x = 18.23, y = 6.78) had an error of only 0.36 compared to the true value (x = 18.01, y = 6.55), which was the smallest error among all compared algorithms and significantly better than them. This indicated that it had a stronger accuracy advantage in dealing with nonlinear correlation and dynamic coupling problems of multi-source sensor data, laying the algorithm foundation for robot localization and mapping in complex dynamic environments. In the performance comparison experiment between dynamic and static scenes, the ESKF-YOLOv5s algorithm showed lower RMSE (0.23) and MSE (0.15) in dynamic scenes compared to other algorithms such as EKF-YOLOv5s (RMSE = 0.25, MSE = 0.18) and YOLOv5s (RMSE = 0.35, MSE = 0.32); In static scenarios, its RMSE (0.12), MSE (0.08), and SSIM (0.95) were all optimal, verifying its reliability in feature extraction and state estimation tasks in different scenarios. In summary, the state estimation algorithm for multi-sensor systems based on ESKF-YOLOv5s has shown outstanding performance in accuracy, scene adaptability, and anti-interference ability, providing a new method for precise positioning, stable navigation, and safe operation of robots in fields such as autonomous driving and special operations.

## 6 Limitations and future work

In summary, there are limitations in the research, such as the limited adaptability of sensor types and the insufficient ability of dynamic object detection to cope with complex scenes. Subsequent research can further expand the types of sensors that can be adapted to provide more comprehensive technical support for multi-sensor state estimation of robots in more complex scenes.

## Supporting information

S1 File Minimal data set definition. (DOC)
