# Supplementary material for: State estimation of multi-sensor systems based on error-state Kalman
Source: PLoS One. 2025 Dec 26;20(12):e0338917. doi: 10.1371/journal.pone.0338917 (PMC12742780; doi:10.1371/journal.pone.0338917)
Supplement: S1 File — (DOC) [file pone.0338917.s001.doc]

**The data in Figure 8 (a)**

| Algorithm | 25 groups | 50 groups | 75 groups | 100 groups | 125 groups | 150 groups |
| --- | --- | --- | --- | --- | --- | --- |
| ESKF-YOLOv5s | 0.33 | 0.28 | 0.25 | 0.22 | 0.20 | 0.20 |
| EKF-YOLOv5s | 0.50 | 0.46 | 0.44 | 0.39 | 0.39 | 0.38 |
| Transformer-LiDAR | 0.41 | 0.35 | 0.34 | 0.34 | 0.31 | 0.29 |
| ESKF-LiDAR | 0.44 | 0.42 | 0.39 | 0.34 | 0.30 | 0.28 |

**The data in Figure 8 (b)**

| Algorithm | 25 groups | 50 groups | 75 groups | 100 groups | 125 groups | 150 groups |
| --- | --- | --- | --- | --- | --- | --- |
| ESKF-YOLOv5s | 0.21 | 0.18 | 0.15 | 0.15 | 0.14 | 0.13 |
| EKF-YOLOv5s | 0.39 | 0.38 | 0.37 | 0.36 | 0.34 | 0.31 |
| Transformer-LiDAR | 0.29 | 0.26 | 0.24 | 0.22 | 0.22 | 0.21 |
| ESKF-LiDAR | 0.33 | 0.28 | 0.27 | 0.26 | 0.25 | 0.24 |

**The data in Figure 9 (a)**

| Algorithm | 15 groups | 30 groups | 45 groups | 60 groups | 75 groups | 90 groups |
| --- | --- | --- | --- | --- | --- | --- |
| ESKF-YOLOv5s | 102.8 | 117.6 | 112.6 | 116.9 | 115.4 | 97.5 |
| EKF-YOLOv5s | 117.5 | 111.2 | 125.1 | 130.3 | 118.8 | 106.9 |
| Actual value | 106.0 | 123.8 | 112.8 | 117.1 | 113.3 | 100.8 |

**The data in Figure 9 (b)**

| Algorithm | 15 groups | 30 groups | 45 groups | 60 groups | 75 groups | 90 groups |
| --- | --- | --- | --- | --- | --- | --- |
| ESKF-YOLOv5s | 107.5 | 117.5 | 85.2 | 100.0 | 97.5 | 102.8 |
| EKF-YOLOv5s | 112.7 | 105.4 | 88.3 | 111.1 | 108.4 | 122.2 |
| Actual value | 112.5 | 119.2 | 81.8 | 99.8 | 89.7 | 102.5 |

**The data in Figure 10 (a)**

| Algorithm | 10 groups | 20 groups | 30 groups | 40 groups | 50 groups | 60 groups |
| --- | --- | --- | --- | --- | --- | --- |
| ESKF-YOLOv5s | 18.8 | 17.9 | 17.9 | 17.5 | 17.0 | 16.9 |
| EKF-YOLOv5s | 21.3 | 20.7 | 20.3 | 20.2 | 20.2 | 20.1 |
| Transformer-LiDAR | 25.2 | 25.1 | 24.8 | 24.7 | 24.3 | 23.8 |
| ESKF-LiDAR | 13.1 | 12.0 | 11.9 | 11.9 | 11.6 | 11.5 |

**The data in Figure 10 (b)**

| Algorithm | 10 groups | 20 groups | 30 groups | 40 groups | 50 groups | 60 groups |
| --- | --- | --- | --- | --- | --- | --- |
| ESKF-YOLOv5s | 16.5 | 15.0 | 14.9 | 14.6 | 14.6 | 14.5 |
| EKF-YOLOv5s | 19.0 | 18.3 | 18.0 | 17.3 | 17.2 | 16.7 |
| Transformer-LiDAR | 23.1 | 22.7 | 22.7 | 22.6 | 22.6 | 21.6 |
| ESKF-LiDAR | 10.8 | 9.6 | 9.2 | 9.1 | 8.7 | 8.9 |

**The data in Figure 11 (a)**

| System | -10dB | -5dB | 0dB | 5dB | 10dB |
| --- | --- | --- | --- | --- | --- |
| ESKF-YOLOv5s | 1.17 | 0.92 | 0.65 | 0.58 | 0.45 |
| EKF-YOLOv5s | 1.39 | 1.17 | 1.11 | 0.98 | 0.77 |
| Transformer-LiDAR | 1.68 | 1.57 | 1.51 | 1.46 | 1.33 |
| ESKF-LiDAR | 1.43 | 1.43 | 1.29 | 1.09 | 0.99 |

**The data in Figure 11 (b)**

| System | -10dB | -5dB | 0dB | 5dB | 10dB |
| --- | --- | --- | --- | --- | --- |
| ESKF-YOLOv5s | 2.07 | 1.87 | 1.31 | 0.95 | 0.66 |
| EKF-YOLOv5s | 2.57 | 2.32 | 1.49 | 1.08 | 0.93 |
| Transformer-LiDAR | 2.85 | 2.47 | 1.73 | 1.24 | 1.21 |
| ESKF-LiDAR | 2.65 | 2.25 | 1.66 | 1.28 | 1.06 |

**The data in Figure 11 (c)**

| System | -10dB | -5dB | 0dB | 5dB | 10dB |
| --- | --- | --- | --- | --- | --- |
| ESKF-YOLOv5s | 0.63 | 0.75 | 0.82 | 0.88 | 0.97 |
| EKF-YOLOv5s | 0.60 | 0.72 | 0.80 | 0.87 | 0.93 |
| Transformer-LiDAR | 0.55 | 0.68 | 0.75 | 0.81 | 0.87 |
| ESKF-LiDAR | 0.58 | 0.69 | 0.79 | 0.83 | 0.90 |

**The data in Figure 12 (a)**

| System | 20 groups | 40 groups | 60 groups | 80 groups | 100 groups | 120 groups |
| --- | --- | --- | --- | --- | --- | --- |
| ESKF-YOLOv5s | 0.030 | 0.028 | 0.025 | 0.024 | 0.021 | 0.020 |
| EKF-YOLOv5s | 0.053 | 0.049 | 0.048 | 0.043 | 0.041 | 0.039 |
| ESKF-LiDAR | 0.081 | 0.078 | 0.075 | 0.074 | 0.066 | 0.065 |

**The data in Figure 12 (b)**

| System | 20 groups | 40 groups | 60 groups | 80 groups | 100 groups | 120 groups |
| --- | --- | --- | --- | --- | --- | --- |
| ESKF-YOLOv5s | 0.060 | 0.051 | 0.053 | 0.044 | 0.036 | 0.033 |
| EKF-YOLOv5s | 0.111 | 0.100 | 0.098 | 0.096 | 0.079 | 0.069 |
| ESKF-LiDAR | 0.126 | 0.122 | 0.113 | 0.102 | 0..094 | 0.094 |

**The data in Figure 13 (a)**

| System | 15 groups | 30 groups | 45 groups | 60 groups | 75 groups | 90 groups |
| --- | --- | --- | --- | --- | --- | --- |
| ESKF-YOLOv5s | 1.12 | 1.04 | 0.99 | 0.69 | 0.69 | 0.53 |
| EKF-YOLOv5s | 2.20 | 2.03 | 1.94 | 1.61 | 1.59 | 1.07 |
| Transformer-LiDAR | 2.92 | 2.86 | 2.47 | 2.35 | 2.15 | 1.80 |
| ESKF-LiDAR | 2.50 | 2.12 | 2.05 | 1.79 | 1.76 | 1.42 |

**The data in Figure 13 (b)**

| System | 15 groups | 30 groups | 45 groups | 60 groups | 75 groups | 90 groups |
| --- | --- | --- | --- | --- | --- | --- |
| ESKF-YOLOv5s | 1.78 | 1.71 | 1.50 | 1.36 | 1.22 | 1.07 |
| EKF-YOLOv5s | 2.40 | 2.34 | 2.30 | 1.98 | 1.71 | 1.65 |
| Transformer-LiDAR | 3.17 | 3.05 | 3.01 | 2.74 | 2.50 | 2.25 |
| ESKF-LiDAR | 2.78 | 2.50 | 2.25 | 1.92 | 1.77 | 1.77 |
